# Is Fibromyalgia Overlooked in Cases of Idiopathic Granulomatous Mastitis? A Cross-Sectional Controlled Study

**DOI:** 10.3390/jcm15197592

**Published:** 2026-09-30

**Authors:** Murat Toprak, Nurşen Toprak

**Affiliations:** 1 Department of Physical Medicine and Rehabilitation, Medical Faculty, Van Yüzüncü Yıl University, Van 65080, Türkiye; 2 Department of Radiology, Medical Faculty, Van Yüzüncü Yıl University, Van 65080, Türkiye

**Keywords:** idiopathic granulomatous mastitis, fibromyalgia, anxiety, depression, fatigue, quality of life

## Abstract

**Introduction**: The purpose of this study was to determine the prevalence of fibromyalgia (FM) syndrome in those with idiopathic granulomatous mastitis (IGM) and to assess the correlations between anxiety, depression, fatigue, sleep quality, and overall quality of life (QOL). **Methods:** Seventy-six patients with idiopathic granulomatous mastitis (IGM) confirmed by tru-cut biopsy and 62 age- and sex-matched non-IGM group were enrolled. Fibromyalgia (FM) was diagnosed according to the American College of Rheumatology criteria. Participants completed the Fibromyalgia Impact Questionnaire-Revised (FIQR), Visual Analog Scale (VAS), Beck Anxiety Inventory (BAI), Beck Depression Inventory (BDI), Functional Assessment of Chronic Illness Therapy–Fatigue (FACIT-Fatigue), Pittsburgh Sleep Quality Index (PSQI), and Short Form-36 Health Survey (SF-36). **Results**: The mean age was similar between the IGM and non-IGM groups (34.25 ± 8.09 vs. 32.54 ± 8.67 years). FM prevalence was significantly higher in patients with IGM than in controls (36.8% vs. 6.5%). Patients with IGM had significantly worse VAS, BAI, BDI, FACIT-Fatigue, PSQI, and FIQR scores (all *p* < 0.001). Within the IGM group, patients with FM had significantly poorer quality of life and worse clinical outcomes than those without FM (all *p* < 0.001). FIQR scores were significantly correlated with quality of life measures. In multivariable analysis, older age and higher BMI were independently associated with lower odds of fibromyalgia, whereas breastfeeding was an independent predictor of fibromyalgia. **Conclusions**: Fibromyalgia is significantly more common in patients with idiopathic granulomatous mastitis than in the non-IGM group and is associated with greater symptom burden and poorer quality of life. Screening for fibromyalgia may improve the comprehensive management of patients with IGM.

## 1. Introduction

Idiopathic granulomatous mastitis (IGM) is a breast condition characterized by non-caseating granulomas, which tend to recur and remit. It mainly affects young women [1]. Kessler and Wolloch first described it in 1972 [2]. Studies suggest that IGM has an autoinflammatory origin; however, the exact cause is unknown [3]. From a clinical and radiological perspective, it may be confused with malignancy [4]. The diagnosis of IGM is histopathological and is made by ruling out other granulomatous diseases with a Tru-Cut biopsy [5]. IGM treatment typically includes immunosuppressive therapies such as corticosteroids and methotrexate (MTX), as well as surgical options [6].

Fibromyalgia (FM) causes pain in the muscles and joints, along with additional symptoms such as sleep disturbances, anxiety, depression, mood disorders, fatigue, and cognitive issues [7]. Current estimates indicate that this syndrome affects 2–8% of the global population, mainly working-age individuals, with a notable gender disparity of 3:1 in favor of women [8]. Its association with rheumatic and autoimmune diseases is prevalent. The cause of fibromyalgia is still not fully understood, but it is believed to arise from multiple factors, including genetic predisposition, immune and neuroendocrine system dysfunction, and environmental stressors. Fibromyalgia significantly affects individuals’ function and quality of life (QOL), leading to socioeconomic impacts on healthcare systems and society [9].

A relapsing-remitting course of progression characterizes both IGM and FM and primarily affects young women. Widespread pain is observed in both, and autoimmunity is important in etiopathogenesis. Given these similarities, is FM more common in IGM patients? Fibromyalgia, a highly prevalent and disabling condition in rheumatology practice, has not previously been systematically assessed in patients with IGM. To address this gap, the present study is the first controlled investigation evaluating the prevalence of fibromyalgia and its clinical impact on pain in patients with IGM. Moreover, while it is clear that fibromyalgia syndrome causes anxiety, depression, and sleep disorders and negatively affects the quality of life of patients, studies on this subject in IGM are limited [10]. IGM is a chronic and benign disease. Still, it can cause significant fear and anxiety in patients due to its resemblance to breast cancer and the requirement for long-term treatment [11]. Furthermore, as treatments can occasionally necessitate surgical intervention (e.g., fistula/abscess drainage, mastectomy), there is a concomitant increase in anxiety and depression.

The study aimed to find out how common fibromyalgia syndrome is among individuals with IGM. Furthermore, the study sought to evaluate the relationships between anxiety, depression, fatigue, sleep quality, and overall QOL.

## 2. Methods

### 2.1. Study Design

Van Yüzüncü Yil University Medical Faculty conducted a study in the Department of Physical Medicine and Rehabilitation (PMR) and the Department of Radiology-Breast in Van, Turkey. The study was conducted from 20 April 2025 to 20 December 2025.

### 2.2. Participant

The present study comprised a total of 76 patients diagnosed with IGM who had undergone Tru-Cut biopsy, as well as 62 age- and sex-matched non-IGM group. The control group, matched for age and sex, was selected from individuals undergoing routine health examinations at our institution during the same study period. A comprehensive questionnaire was administered to all participants, encompassing inquiries into their age, height, weight, the duration of their disease, the treatments they had undergone, breastfeeding, parity, smoking status, BI-RADS (The Breast Imaging Reporting and Data System) staging, and the presence of any secondary diseases and their duration, if applicable.

### 2.3. The Eligibility Criteria

Patients aged 18+ with a confirmed IGM diagnosis by tru-cut biopsy took part in the study. People with rheumatic diseases, infectious diseases, cancers, liver or kidney disorders, systemic diseases like unregulated diabetes, thyroid problems, or psychiatric disorders, and people who had taken psychiatric meds in the last 3 months were not part of this study. Also, the non-IGM control group consisted of individuals who underwent routine clinical examinations during the study period and had no history or diagnosis of IGM.

### 2.4. Assessments

The diagnosis was made following the American College of Rheumatology’s FM criteria [12]. A diagnosis of FM is established when the following three conditions are met, according to the ACR 2016 criteria: 1. The Widespread Pain Index (WPI) must be seven or higher, and the Symptom Severity Scale (SSS) score must be five or higher; alternatively, the WPI must be between 4 and 6, with an SSS score of 9 or higher. 2. The generalized pain is present in at least four of five regions: upper left and right, lower left and right, and the axial skeleton. 3. Symptoms present for >3 months. Participants were categorized as either having or not having FM using the WPI and the SSS.

#### 2.4.1. Visual Analog Scale (VAS)

Pain intensity was evaluated using a 10 cm Visual Analog Scale (VAS), where 0 represented “no pain” and 10 represented “the worst imaginable pain” [1]. Participants marked a point along the line that best reflected their current pain level. Participants marked a point along the line that best reflected their current pain level.

#### 2.4.2. Beck Anxiety Inventory (BAI)

Anxiety levels were assessed using the Beck Anxiety Inventory (BAI), a 21-item self-report questionnaire. Each item is scored on a 4-point Likert scale ranging from 0 (“not at all”) to 3 (“severely”), yielding a total score between 0 and 63. Higher total scores indicate higher levels of anxiety. The Turkish validity and reliability study of the BAI was conducted by Ulusoy et al. [13].

#### 2.4.3. Beck Depression Inventory (BDI)

Depressive symptoms were evaluated using the Beck Depression Inventory (BDI), which consists of 21 self-report items. Each item is rated from 0 to 3, with the total score ranging from 0 to 63. Higher scores reflect greater severity of depressive symptoms. The Turkish adaptation, validity, and reliability of the BDI were established by Hisli [14].

#### 2.4.4. Pittsburgh Sleep Quality Index (PSQI)

Sleep quality over the past month was evaluated using the Pittsburgh Sleep Quality Index (PSQI). It comprises 19 self-rated items generating seven component scores (subjective sleep quality, sleep latency, sleep duration, habitual sleep efficiency, sleep disturbances, use of sleep medication, and daytime dysfunction). Each component is scored from 0 to 3, resulting in a global score ranging from 0 to 21, where scores > 5 indicate poor sleep quality. The Turkish validity and reliability of the PSQI were demonstrated by Agargun et al. [15].

#### 2.4.5. Functional Assessment of Chronic Illness Therapy—Fatigue (FACIT-F)

Fatigue severity and its functional impact were measured using the Functional Assessment of Chronic Illness Therapy—Fatigue (FACIT-F) scale. This 13-item tool uses a 5-point Likert scale (0 = “not at all” to 4 = “very much”). Total scores range from 0 to 52, with higher scores representing lower fatigue and better functional status. The Turkish validation of the FACIT-F was performed by Cinar et al. [16].

#### 2.4.6. Short Form 36 (SF-36)

Health-related quality of life was assessed using the Short Form-36 (SF-36) health survey. It contains 36 items divided into eight domains: physical functioning, role physical, bodily pain, general health, vitality, social functioning, role emotional, and mental health. Scores for each domain range from 0 to 100, with higher scores indicating better health status. The Turkish adaptation, validity, and reliability were established by Kocyigit et al. [17].

#### 2.4.7. Revised Fibromyalgia Impact Questionnaire (FIQR)

Disease severity and functional impact in fibromyalgia patients were evaluated using the Revised Fibromyalgia Impact Questionnaire (FIQR). The FIQR includes 21 items across three domains: function, overall impact, and symptoms. Each item is scored on an 11-point numeric scale (0 to 10), yielding a total score from 0 to 100, where higher scores reflect greater disease burden. The Turkish cross-cultural adaptation and validation of the FIQR were conducted by Ediz et al. [18].

### 2.5. Statistical Analysis

Statistical analyses were performed using IBM SPSS Statistics for Windows, version 23.0 (IBM Corp., Armonk, NY, USA). The distribution of continuous variables was thoroughly assessed using the Shapiro–Wilk test, along with a review of skewness and kurtosis values, as well as visual inspections of histograms and Q-Q plots. Descriptive statistics were presented as mean ± standard deviation (SD) for data that followed a normal distribution. In contrast, the median and interquartile range (IQR), or the minimum and maximum values, were used for variables that did not follow a normal distribution. Categorical data were expressed as frequencies and percentages. For the comparison of continuous independent variables between two groups, Student’s *t*-test was employed for parametric data, and the Mann–Whitney U test was utilized for non-parametric data. Levene’s test was applied to assess variance homogeneity. Categorical variables were analyzed using the Pearson Chi-square test or Fisher’s exact test when the expected cell counts were less than five. Correlations between psychological scores and clinical parameters were evaluated using Spearman’s rank correlation coefficient, suitable for non-linear or non-parametric distributions. All statistical tests were two-tailed, and a *p*-value < 0.05 was considered to indicate statistical significance. Results were reported with 95% confidence intervals (CIs) to indicate the precision of the estimates. A multivariate logistic regression analysis was performed exclusively in the IGM group to identify independent factors associated with the presence of fibromyalgia among patients with idiopathic granulomatous mastitis. Results of the regression analysis were presented as odds ratios (OR) with 95% confidence intervals (CI). Additionally, to evaluate the potential confounding effect of the wide age range, age-adjusted analyses (Analysis of Covariance [ANOVA] for parametric variables, ordinal logistic regression for non-parametric variables, and partial correlation analysis) were conducted as secondary sensitivity analyses.

### 2.6. Ethics Approval

The study protocol received approval from the Ethics Committee at Van YY University Faculty of Medicine Hospital. The decision number for this study is B.30.2.YYU.0.0.00.00/07 (Decision No: 05). The research was carried out in compliance with the principles of the Declaration of Helsinki. All participants received detailed information about the study and voluntarily gave written informed consent prior to enrolment.

## 3. Results

“Of the initial 85 patients assessed for eligibility, 9 were excluded: 5 because of missing data and 4 because they did not complete the standardized psychological assessment protocol. Consequently, 76 patients were included in the final analysis.” Patients with IGM averaged 34.25 ± 8.09 (range:23–63) years, while non-IGM controls averaged 32.54 ± 8.67 (range:22–63) years, with no significant difference (*p* = 0.090). IGM demographic/clinical characteristics, as well as those of a control group, are outlined in Table 1.

**Figure 1:**
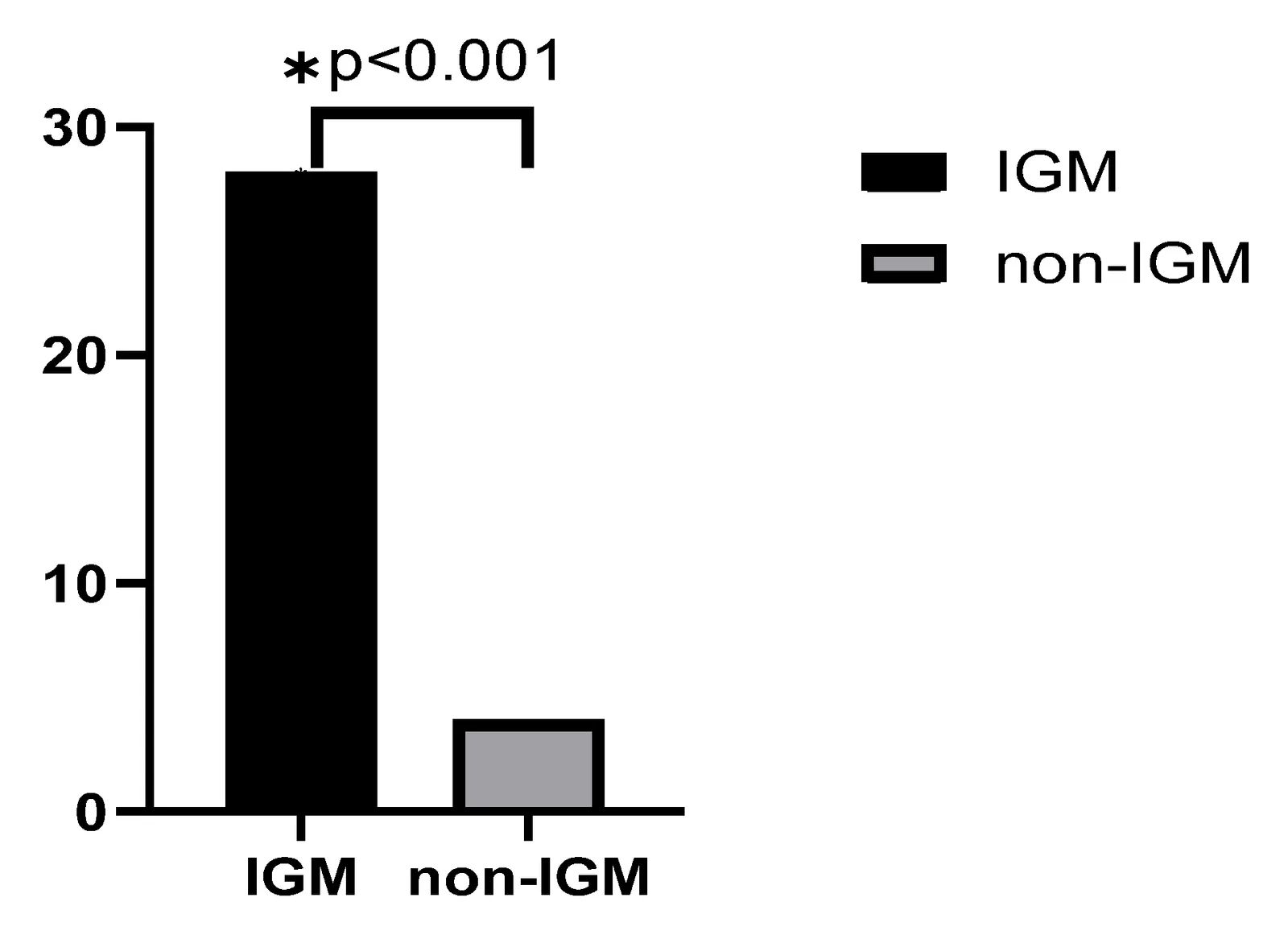
Frequency of FM in the IGM and non-IGM control groups. FM was diagnosed according to the 2016 American College of Rheumatology criteria. FM: fibromyalgia, IGM: idiopathic granulomatous mastitis; * *p* < 0.001.

A significantly higher frequency of fibromyalgia (FM) was found in the IGM group (28 patients, 36.8%) compared to the non-IGM control group (4 patients, 6.5%), with a *p*-value of less than 0.001 (Figure 1).

Significant differences were noted between the IGM and non-IGM control groups in their VAS, BAI, BDI, FACIT, PSQI, and FIQR scores (all *p* < 0.001). All results are displayed in Table 2.

In patients with IGM, those who underwent abscess drainage or surgery (*n* = 12) had lower mean scores on the Beck Anxiety Inventory (BAI) (7.17 ± 3.76) and Beck Depression Inventory (BDI) (7.33 ± 3.96) compared to those receiving other medical treatments (*n* = 64) with scores of 9.64 ± 4.09 and 9.36 ± 4.04, respectively. However, due to the small sample size for invasive procedures, these findings should be interpreted cautiously, as they did not reach statistical significance (BAI, *p* = 0.066; BDI, *p* = 0.080).

The scores for VAS, BAI, BDI, FACIT, and PSQI showed significant differences between patients with fibromyalgia and those without (*p* < 0.001) (Table 3).

IGM patients with FM had significantly lower quality of life scores (for all, *p* < 0.001) (Table 4).

Correlation analysis revealed statistically significant moderate negative correlations between FIQR scores and all domains of the SF-36 health survey (r: −0.402 to −0.417, all *p* < 0.05), indicating that higher fibromyalgia impact was consistently associated with poorer health-related quality of life across physical, emotional, and social domains. Conversely, no statistically significant correlations were observed between FIQR scores and VAS (r = 0.035, *p* = 0.857), BAI (r = 0.266, *p* = 0.170), BDI (r = 0.054, *p* = 0.781), FACIT (r = −0.167, *p* = 0.393), or PSQI (r = 0.234, *p* = 0.229). Table 5 shows the correlation of FIQR scores with other study parameters.

Multivariate logistic regression analysis was performed only in patients with idiopathic granulomatous mastitis to determine independent predictors of fibromyalgia within the IGM cohort. Only age, breastfeeding, and BMI remained statistically significant in the multivariate analysis model, while marital status, disease duration, and gravidity were not independent predictors of fibromyalgia (Table 6).

## 4. Discussion

We evaluated IGM patients for fibromyalgia, anxiety, depression, fatigue, and sleep quality, using BAI, BDI, FACIT, PSQI, SF-36, and FIQR. FM was more prevalent in IGM patients than in the non-IGM control group, with an increase observed in pain, anxiety, depression, and fatigue scores. Patients with IGM had significantly higher PSQI scores than controls, indicating poorer sleep quality in the IGM group. Moreover, significant increases were observed in pain, anxiety, depression, fatigue, and FIQR scores in IGM patients with FM, compared to those without FM. Quality-of-life scores in the study group were significantly lower than those in the non-IGM control group. This decline was observed across all QOL subgroups. These results indicate that IGM is associated with fibromyalgia, anxiety, depression, fatigue, and reduced QOL.

FM is a disease marked by widespread pain, anxiety, depression, sleep disturbances, and fatigue. FM is a prevalent condition in clinical settings and is recognized as a substantial cause of morbidity that requires treatment on a global scale. FM prevalence varies by age, sex, and country. In Turkey, the prevalence of FM syndrome in adults is estimated at 2–8%, with a global mean prevalence of 2.7%. The FM frequency in the non-IGM control group (6.5%) matched that reported in previous studies [8,19]. FM was found in 36.8% of the study group, indicating a high prevalence. This disease, which hurts quality of life, has not been studied enough in specific patient populations, such as IGM patients. To our knowledge, this is the first study to examine FM in patients with IGM.

Pain, mass, discharge, hardness, and redness in the breast area are common in patients with IGM. Based on these symptoms, it has been suggested that patients may have FM as a result of systemic symptoms such as anxiety, depression, fatigue, and widespread pain. Furthermore, given the shared pathophysiological features of IGM and FM, such as autoimmunity and inflammation, the fact that both diseases occur at a young age, and especially the female predominance, we investigated the frequency of co-occurrence of these two diseases. Moreover, the literature shows that IGM is not merely a localized breast disease but a complex condition affecting patients’ psychosocial lives, sleep patterns, and quality of life.

An important consideration is whether FM symptoms observed in patients with IGM represent coexisting primary FM or a secondary FM-like state associated with the chronic pain and inflammatory burden of IGM. Persistent nociceptive input, psychological distress, sleep disturbances, and inflammation-related central sensitization may potentially contribute to the development or amplification of widespread pain and other FM-related symptoms. However, given the cross-sectional design of our study, the temporal and causal relationship between IGM and FM cannot be determined. In particular, we could not establish whether FM preceded the onset of IGM or developed subsequently as a consequence of the chronic disease burden. Longitudinal studies assessing FM status before and after IGM onset and following disease remission are required to distinguish primary FM from a potential secondary FM phenotype.

The association reported in the literature between mastalgia and chronic pain syndromes suggests that fibromyalgia-like symptoms may coexist with or be exacerbated by chronic breast pain in patients with IGM [20,21,22]. Previous studies have reported alterations in inflammatory mediators, including interleukin-6 (IL-6), tumor necrosis factor-α (TNF-α), and interleukin-1β (IL-1β), in inflammatory conditions and in patients with FM. These findings have led to hypotheses regarding potentially shared inflammatory or immunological pathways between IGM and FM. However, no cytokines, inflammatory mediators, or immune biomarkers were measured in the present study. Therefore, these proposed mechanisms cannot be directly evaluated or confirmed from our data. The observed higher prevalence of FM among patients with IGM should consequently not be interpreted as evidence that inflammation mediates or causes FM. Rather, shared inflammatory, pain-related, and psychosocial mechanisms may represent potential biological hypotheses that require confirmation in longitudinal studies incorporating direct immunological and biomarker assessments.

Chronic pain and inflammation associated with IGM may theoretically contribute to mechanisms involved in central sensitization, which is an important feature of FM. However, this proposed relationship remains hypothetical, and our cross-sectional study cannot establish whether chronic pain or inflammation related to IGM contributes to the development or amplification of FM symptoms. Additionally, the literature on mastalgia in patients with FM suggests that breast pain may involve overlapping nociplastic pain mechanisms [22]. Shared mechanisms involving stress reactivity and hypothalamic–pituitary–adrenal (HPA) axis activity have also been proposed as potential links between IGM and FM. These mechanisms should be regarded as possible biological explanations for the observed coexistence rather than causal pathways demonstrated by the present study. Although IGM and FM may share inflammatory, immunological, and pain-related features, the precise mechanisms underlying their coexistence remain unclear [23].

Emotional disorders, particularly anxiety and depression, are frequently observed in patients with chronic inflammatory diseases and may substantially impair quality of life. Emotional dysfunction is also recognized as an important component of the disease burden in rheumatic and other chronic inflammatory conditions. Inflammatory cytokines may contribute to psychological symptoms through bidirectional interactions between the immune, neuroendocrine, and central nervous systems. Proinflammatory cytokines, including IL-1β, IL-6, and TNF-α, can influence hypothalamic–pituitary–adrenal (HPA) axis activity, neurotransmitter metabolism, and neural plasticity, potentially contributing to anxiety- and depression-related symptoms. In IGM, dysregulation of several inflammatory mediators, including IL-6, IL-8, IL-17, IL-22, and IL-23, has been reported. Therefore, inflammation-related neuroimmune mechanisms may contribute to the psychological burden observed in patients with IGM [24,25]. Therefore, psychological vulnerability in fibromyalgia is likely multidimensional rather than reducible to anxiety and depression alone, as recent evidence suggests that catastrophizing, anxiety, and depressive symptoms are interrelated but may also show partly distinct biological associations [26].

However, based on the information we have, it is unclear if the deterioration in IGM patients is the cause or the result. Kehribar et al. [10] reported an increase in anxiety and depression levels in young women diagnosed with IGM. The presence of fistulas, bilateral lesions, and the duration of the disease were linked to psychological symptoms. The cross-sectional study involved a small sample size. It is important to note that fibromyalgia may not exclusively mediate the high rates of depression and pain observed in our cohort. While FM represents a significant comorbidity, the chronic nature of IGM itself—characterized by recurrent abscesses and long-term steroid use—contributes independently to psychological morbidity. Unlike patients with primary FM (without IGM), our patients face an additional burden of localized physical disfigurement and inflammatory flares, which may exacerbate central pain sensitization.

Potential confounding effects of medical treatments—specifically systemic corticosteroids, which are known to influence mood and sleep architecture—were also considered. However, our subgroup comparisons within the IGM cohort revealed no significant disparities in anxiety, depression, or sleep quality scores between patients receiving corticosteroid therapy and those managed without steroids. This suggests that the psychological and sleep impairments observed in the IGM group are primarily driven by the chronic inflammatory and painful nature of the disease and fibromyalgia comorbidity rather than pharmacological exposure.

IGM patients with FM comorbidity had higher BAI, BDI, and PSQI scores than those without FM. Additionally, the FIQR score was linked to the BAI, BDI, and PSQI scores.

FACIT scores were significantly lower in the IGM group than in non-IGM controls (*p* < 0.001), indicating that individuals in the IGM group reported higher levels of fatigue. Moreover, FACIT scores were lower among IGM patients with FM compared to those without FM.

Patients with IGM and FM comorbidity exhibited poorer quality of life in all SF-36 domains than patients without FM. The FIQR score showed strong correlations with all SF-36 domains. Notably, patients with FM report lower QOL than those with other conditions and the general population [2,27,28]. This is the first study so far to investigate the effect of FM on QOL in IGM patients.

In multivariable logistic regression analysis, older age (OR = 0.943) and higher BMI (OR = 0.868) were associated with lower odds of fibromyalgia, although these associations should be interpreted cautiously given their modest effect sizes and the observational nature of the study.

This study had several limitations. First, it was conducted at a single center and included a relatively small number of participants. Second, we did not compare patients who relapsed with those in remission. Third, we used the PSQI to assess sleep quality, but a polysomnography assessment could have provided more reliable results. Fourth, this study did not include a control group consisting of patients with Fibromyalgia but without IGM. Such a comparison would be valuable in determining whether the pain–depression axis in IGM patients differs qualitatively or quantitatively from the general FM population. A key limitation of this study is the exclusion of patients with a history of psychiatric disorders or recent use of psychotropic medications. While this was intended to focus on the psychological impact of IGM, it likely introduces selection bias. Therefore, our findings may not accurately reflect the overall prevalence of anxiety and depression in the IGM population. Another limitation is that assessor blinding was not feasible because participants’ group assignments were generally apparent from their clinical history and examination findings. Although standardized and validated assessment instruments were used, the lack of blinding may have introduced observer or expectation bias during the assessment of fibromyalgia and psychological symptoms. Future multicenter studies with broader inclusion criteria are needed to provide a more comprehensive view.

## 5. Conclusions

In conclusion, our study indicates a clinically noteworthy prevalence of FM criteria among IGM participants alongside lower quality of life scores. However, considering the single-centre, cross-sectional design and potential confounding factors, these findings primarily support increased clinical awareness of FM symptoms in patients presenting with IGM. Further prospective and multi-centre studies are warranted to evaluate the utility of targeted FM screening in this population.

## Figures and Tables

**Table 1:** Demographic and clinical characteristics of patients with IGM and non-IGM controls.

Characteristics	IGM (*n*= 76)	Non-IGM (*n* = 62)	*p*
Age, years, mean ± SD	34.25 ± 8.09	32.54 ± 8.67	0.090
Height, cm	160.89 ± 5.15	160.89 ± 5.48	0.191
Weight, kg	71.09 ± 8.99	70.38 ± 9.57	0.692
BMI, (kg/m^2^)	27.21 ± 3.47	27.02 ± 3.65	0.896
Duration of disease, months, mean ± SD	37.55 ± 29.08		
Lesion side, *n* (%)			
Right	31 (40.8%)		
Left	32 (42.1%)		
Bilateral	13 (17.1%)		
Breastfeeding history, *n* (%)	69 (90.8%)	39 (62.9%)	<0.001
Number of pregnancies, mean ± SD	2.67 ± 1.18	1.96 ± 0.81	<0.001
Smoking, current, *n* (%)	18 (23.7%)	17 (27.4)	0.379
ESR, mm/h	17.07 ± 14.11	7.06 ± 2.98	<0.001
CRP, mg/dL	9.22 ± 11.86	5.87 ± 3.72	<0.001
Treatments, *n* (%)			
Steroids	26 (34.2%)		
Steroids + Antibiotics	6 (7.9%)		
Steroids + Abscess drainage	5 (6.6%)		
Steroids + NSAID	4 (5.3%)		
Antibiotics	6 (7.9%)		
Antibiotics + Abscess drainage	5 (6.6%)		
Surgery	7 (9.2%)		
Untreated	17 (22.3%)		
Radiological finding, *n* (%)			
Ultrasound finding			
Hypoechoic mass	15 (19.7%)		
Collection/abscess	59 (77.6%)		
Skin thickening	20 (26.3%)		
Axillary lymphadenopathy	41 (53.9%)		
Fistulae/sinus tract	35 (46.1%)		
BI-RADS			
3	14 (18.4%)		
4a	18 (23.7%)		
4b	21 (27.6%)		
4c	17 (22.4%)		
5	6 (7.9%)		
Elastography finding			
ARFI elastography, mean ± SD	3.21 ± 1.13		
MRI finding			
Non-mass enhancement	58 (76.3%)		
Rim enhancement	57 (75%)		
Abscess	60 (78.9%)		
Skin thickening	21 (27.6%)		
Fistula/sinus tract	37 (48.7%)		
FM fequency, *n*(%)	28 (36.8%)	4 (6.5%)	<0.001
WPI (IGM patients with FM), mean ± SD	10.09 ± 3.52		
SSS (IGM patients with FM), mean ± SD	6.11 ± 2.41		

IGM: idiopathic granulomatous mastitis, BMI: Body mass index; CRP: C-reactive protein; ESR: Erythrocyte sedimentation rate; NSAID: Non Steroidal Anti-Inflamatory Drug; BI-RADS: The Breast Imaging Reporting and Data System; FM: Fibromyalgia; WPI: Widespread Pain Index; SSS: Symptom Severity Scale; SD: standard deviation.

**Table 2:** Comparison of VAS, BAI, BDI, FACIT, PSQI, and FIQR scores between patients with IGM and non-IGM control group.

	IGM (*n* = 76)		Non-IGM (*n* = 62)		*p*
	Median	(IQR)	Median	(IQR)	
VAS	9	(8–10)	5	(3–6)	<0.001
BAI	9	(6–12)	7	(5–11)	<0.001
BDI	9	(7–17)	7	(5–8)	<0.001
FACIT	26	(18–28)	45	(42–48)	<0.001
PSQI	8	(6.25–9)	4	(3–5)	<0.001
FIQR	45	(40–71.5)	35	(30–40)	<0.001

IGM: idiopathic granulomatosis mastitis; IQR: interquartile range, VAS: Visual Analog Scale; BAI: Beck Anxiety Inventory; BDI: Beck Depression Inventory; FACIT: Functional Assessment of Chronic Illness Therapy; PSQI: Pittsburgh Sleep Quality Index; FIQR: Revised Fibromyalgia Impact Questionnaire.

**Table 3:** A comparison between IGM patients with and without FM.

	IGM with FM (*n* = 28)		IGM Without FM (*n* = 48)		*p*
	Median	(IQR)	Median	(IQR)	
VAS	9	(8–10)	7	(6–9)	<0.001
BAI	12	(8–15)	7	(5–11)	<0.001
BDI	14	(7–17)	7	(6–9)	<0.001
FACIT	17	(15–21)	27	(25–29)	<0.001
PSQI	10	(7–12)	8	(6–9)	<0.001
FIQR	82	(70–90)	40	(31–45)	<0.001

IGM: idiopathic granulomatosis mastitis; IQR: interquartile range; FM: Fibromyalgia; VAS: Visual Analog Scale; BAI: Beck Anxiety Inventory; BDI: Beck Depression Inventory; FACIT: Functional Assessment of Chronic Illness Therapy; PSQI: Pittsburgh Sleep Quality Index; FIQR: Revised Fibromyalgia Impact Questionnaire.

**Table 4:** Quality of life between IGM patients with and without fibromyalgia (FM) according to Short Form-36.

SF-36	IGM with FM (*n* = 28)	IGM Without FM (*n* = 48)	*p*
Physical Function	31.25 ± 5.71	60.10 ± 0.72	<0.001
Role—Physical	41.25 ± 5.71	70.16 ± 1.15	<0.001
Role—Emotional	31.07 ± 5.67	63.10 ± 1.72	<0.001
Energy-Vibrancy	41.42 ± 7.75	79.79 ± 1.44	<0.001
Mental health	31.60 ± 7.58	70.10 ± 1.18	<0.001
Social Functioning	21.60 ± 7.47	60.09 ± 2.78	<0.001
Pain	31.42 ± 7.56	70.13 ± 1.96	<0.001
General health	41.25 ± 7.65	80.11 ± 0.69	<0.001

Values are expressed as mean ± standard deviation. IGM: idiopathic granulomatosis mastitis; FM: fibromyalgia.

**Table 5:** Correlation of Revised Fibromyalgia Impact Questionnaire (FIQR) scores with other variables in patients with IGM.

	FIQR	
	r	*p*
VAS	0.035	0.857
BAI	0.266	0.170
BDI	0.054	0.781
FACIT	−0.167	0.393
PSQI	0.234	0.229
Physical Function	−0.402	0.033
Role—Physical	−0.411	0.034
Role—Emotional	−0.415	0.028
Energy-Vibrancy	−0.413	0.028
Mental health	−0.406	0.031
Social Functioning	−0.407	0.031
Pain	−0.415	0.026
General health	−0.417	0.027

IGM: idiopathic granulomatosis mastitis; VAS: Visual Analog Scale; BAI: Beck Anxiety Inventory; BDI: Beck Depression Inventory; FACIT: Functional Assessment of Chronic Illness Therapy; PSQI: Pittsburgh Sleep Quality Index; FIQR: Revised Fibromyalgia Impact Questionnaire.

**Table 6:** Multivariate Logistic Regression Analysis Identifying Independent Predictors of Fibromyalgia Among Patients with Idiopathic Granulomatous Mastitis (IGM).

Variables	β (B)	SE	Wald	*p*-Value	Odds Ratio (OR)	95% CI for OR
Age, years	−0.059	0.029	5.289	0.021	0.943	0.897–0.991
BMI kg/m^2^	−0.141	0.066	4.555	0.033	0.868	0.763–0.989
Marital status	−0.101	0.097	1.084	0.305	0.904	0.748–1.092
Breastfeeding	1.779	0.503	12.533	<0.001	5.924	2.212–15.863
Disease duration (months)	0.008	0.009	0.811	0.368	1.008	0.991–1.025
Gravidity	0.064	0.052	1.507	0.227	1.066	0.963–1.180

IGM: idiopathic granulomatosis mastitis; BMI: Body Mass Index; SE: Standard Error; CI: Confidence Interval.

## Data Availability

The data that support the findings of this study are available from the corresponding author upon reasonable request.
